# Supplementary material for: Preparedness and response to community spread of COVID-19 governmental and national recommendations for COVID-19 by the Pan-Academic Action Committee
Source: Epidemiol Health. 2020 Apr 29;42:e2020020. doi: 10.4178/epih.e2020020 (PMC7285445; doi:10.4178/epih.e2020020)
Supplement: Supplementary file 1 [file epih-42-e2020020-suppl.pdf]

Volume: 42, Article ID: e2020020
https://doi.org/10.4178/epih.e2020020

「코로나바이러스감염증-19 지역사회 확산」 대비・대응 범학계 코로나바이러스감염증-19 대책위원회 대정부・국민 권고안

코로나19 지역사회 확산 피해 최소화를 위해 전 사회적 역량을 모아주시길 바랍니다

지역사회 전파 차단을 염원하는 우리의 바램과 방역당국의 총력 봉쇄에도 불구하고 코로나바이러스감염증-19(코로나19)의 지역사회 확산은 현실로 다가왔습니다. 그러나 너무 두려워할 필요는 없습니다. 다행히 코로나19의 정체가 속속 밝혀짐에 따라 이제부터는 더욱 정교한 대응도 가능해지고 있습니다. 코로나19는 지금 전 세계적으로 전파되고 있는 새로운 감염병이지만, 인류의 역사에서 새로운 감염병의 도전으로부터 자유로웠던 적은 없었습니다. 우리는 코로나19보다 훨씬 치명적인 사스(SARS)와 메르스(MERS)를 극복하였으며, 코로나19보다 훨씬 전염력이 높은 신종플루도 백신을 개발하여 잘 통제하고 있습니다.

지금까지 밝혀진 코로나19의 임상적 특성을 종합하면, 2월 20일 현재 중국의 후베이성은 3.3%의 치명률을 보이지만 후베이성 이외 지역(0.7%)과 중국 외 발생 국가(0.9%)는 1% 미만의 치명률을 보이고 있습니다. 이는 인플루엔자의 치명률 0.05%보다 높지만, 사스의 10%, 메르스의 30%보다는 크게 낮습니다. 그러나 코로나19는 초기 증상이 심하지 않아 일반 감기와 유사하고, 이 시기에 바이러스의 배출량이 많아 결과적으로 지역사회 전파가 아주 빠르고 높은 것으로 관찰되고 있습니다. 특히 노인과 만성질환자와 같은 취약집단에서는 위중한 결과를 초래할 수 있습니다. 중국의 보고에서도 60세 이상의 고령자가 전체 환자의 30%와 사망의 80% 이상을 차지하고 있습니다.

이렇게 증상은 경미하면서, 전염력이 높은 새로운 바이러스 감염병의 지역사회 전파를 완벽히 차단하는 것은 사실상 불가능합니다. 이는 지금 25여개 국가에서 환자 발생이 보고되고, 중국뿐 아니라 인근 홍콩, 일본, 싱가포르 등에서 지역사회 감염이 확산되는 현황에서도 확인할 수 있습니다. 따라서 이제는 확진자 발견과 접촉자 격리 등 차단 중심의 봉쇄전략(1차 예방)에서 지역사회 확산을 지연시키고, 이로 인한 건강피해를 최소화하는

Volume: 42, Article ID: e2020020
https://doi.org/10.4178/epih.e2020020

완화전략(2차 예방)의 방향으로 전환해 나가야 할 시기가 되었다고 할 수 있습니다.

즉 지역사회 감염 확산이라는 변화된 상황에 맞게 방역의 목표와 전략을 수정해나가야 합니다. 이에 우리 '범학계 코로나19 대책위원회'에서는 정부와 국민들에게 다음과 같은 제언을 드립니다.

정부는,

첫째, 지금까지의 봉쇄전략에서 효율적인 피해 최소화 (완화)전략으로의 이행을 위해 필요한 조치와 방안을 점검하고, 특히 지역사회 차원의 통합방역활동체계가 강화될 수 있도록 빠르게 조치를 시행해 주기 바랍니다.

둘째, 지역사회로 확산된 코로나19 대응을 위한 **비상 의료전달체계를 시급히 마련**하여 주기 바랍니다. 이를 통해 일선 의료기관의 정상적 진료활동이 위축되지 않도록 해 주기 바랍니다. 노출원을 파악해서 접촉자 관리에 집중하는 기존 전략은 현재 상황에서 효과를 기대하기 어려운 상황입니다. 강력한 이동제한과 함께 구체적인 의료시스템 구축이 필요합니다. **고위험 환자들이 일반 의료기관에서 안전하게 치료를 받을 수 있도록 발열-호흡기 선별진료를 전담하는 의료기관과 코로나19 치료병원 지정 및 지원을 촉구합니다.** 아울러 응급환자, 노인, 만성질환 환자의 진료 공백이 생기지 않도록 구체적이고 실효성 있는 건강보호대책을 수립해 주기 바랍니다.

셋째, 정확한 상황 판단을 위한 정보를 국민들과 빠르게 공유하며 방역당국에 대한 신뢰가 제고될 수 있도록 **위기소통활동을 대폭 강화**하여야 합니다. 이를 위해 방역당국, 감염병 전문가, 지역사회 주민들과의 상시적 대화 채널을 다각적으로 만들기 바랍니다. 이를 통해 지역주민이 불안에 위축되기보다 자신들이 거주하는 지역에서 감염확산 방지에 동참하고, 보다 적극적으로 나설 수 있도록 해 주기 바랍니다.

넷째, 열, 호흡기 증상 등이 있는 아이들이나 학생, 직장인은 **진단서가 없어도 공결이나 병가를 쓸 수 있도록** 하고, **아픈 아이를 돌보기 위해 부모가 병가를 쓰는 것으로 인한 불이익이 생기지 않도록 정부와 기업이 지원**하는 방안을 마련해 주기 바랍니다.

Volume: 42, Article ID: e2020020
https://doi.org/10.4178/epih.e2020020

끝으로, 인권이 곧 방역임을 인식하고, **취약계층, 외국인, 장애인 등도 코로나19 진료를 받는 데 어려움이 없도록** 조치하여 주기 바랍니다.

국민 여러분,

신종 바이러스로 인한 또 한 번의 위기가 우리 사회의 정상적 경제활동을 심각하게 위축시키고, 우리 모두의 일상을 불안하게 하고 있습니다. 그러나, 모든 국민들이 함께 노력한다면 이 어려움을 충분히 이겨낼 수 있습니다. 이를 위해 진료현장의 의료진과 지역의 방역 인력은 최선을 다하고 있습니다. 이들의 열정과 노력을 믿고 성원해 주시기 바라면서, 국민 여러분께 다음과 같은 당부 말씀을 드리고자 합니다.

첫째, 평소 손을 자주, 비누로 30초 이상 꼼꼼하게 씻고, 기침이나 재채기를 할 때 입과 코를 휴지나 옷소매로 가리고 하는 등 **개인위생을 철저히** 지켜주시기 바랍니다.

둘째, 열, 기침이나 목아픔, 코막힘이나 콧물 등의 가벼운 **호흡기 증상이 나타나면 외출을 자제해주시고 일반감기약을 드시면서 4-5일 경과를 관찰하시기 바랍니다. 38도 이상 고열이 지속되거나 증상이 심해지거나 계속된다면 진료** 받으시길 바랍니다. 증상이 계속된다면, 가까운 선별진료소를 방문하거나 관할 보건소, 1339에 상담을 요청하시기 바랍니다. 증세가 가벼운 환자는 반드시 큰 병원에 가지 않으셔도 됩니다. **큰 병원은 중증환자 진료에 집중하도록 협조**하여 주시기 바랍니다. 증상이 경미해도 증상 발생 초기에 전파가 이루어질 수 있으므로 증상 발생 이후 5일 이상 외출을 삼가야 합니다. 이에 따른 결석이나 병가를 학교와 직장에서 충분히 보장해주시기 바랍니다.

셋째, **만성질환이 있거나 65세 이상 어르신**이라면 감염에 더 취약할 수 있습니다. 평소 **여러 사람들이 모이는 장소에 출입을 삼가시고 외출 시, 마스크를 착용**하시기 바랍니다.

넷째, 학생과 학부모, 그리고 교사 여러분께 당부드립니다. **학생들 중에서 열, 호흡기 증상이 있는 경우 등교 중지 조치**를 취하시기 바랍니다. 아직 추운 날씨이지만 학생들이 흐르는 물에 비누로 **수시로 손을 씻을 수 있도록 시설을 점검하고 준비**해주시기 바랍니다.

끝으로, 지역사회 확산을 막고자 하는 **의료진과 방역당국의 조치에 적극적으로 따라**

Volume: 42, Article ID: e2020020
https://doi.org/10.4178/epih.e2020020

주시길 바랍니다. 지금과 같이 지역사회 감염이 확산되는 시기에 극히 소수라도 일탈 행위가 반복된다면 그 어떤 방역망으로도 이 위기를 이겨내기 어렵습니다.

우리 모두 한마음으로, 차분하게, 전 사회적 역량을 모아 이 어려움을 극복해 나아갑시다!!

2020년 2월 22일

범학계 코로나바이러스감염증-19 대책위원회

대한감염학회 대한결핵및호흡기학회 대한소아감염학회 대한예방의학회 대한응급의학회 대한의료관련감염관리학회 대한중환자의학회 대한항균요법학회 한국역학회
